# The effectiveness of hot-air, infrared and hybrid drying techniques for lemongrass: appearance acceptability, essential oil yield, and volatile compound preservation

**DOI:** 10.1038/s41598-023-44934-6

**Published:** 2023-11-01

**Authors:** Roghayeh Setareh, Khosro Mohammadi-Ghermezgoli, Hossein Ghaffari-Setoubadi, Saeideh Alizadeh-Salteh

**Affiliations:** 1 Department of Biosystems Engineering, Faculty of Agriculture, University of Tabriz, Tabriz, Iran; 2 Department of Horticultural Science, Faculty of Agriculture, University of Tabriz, Tabriz, Iran

**Keywords:** Analytical biochemistry, Chemical engineering, Biomedical engineering, Secondary metabolism

## Abstract

Lemongrass is a fragrant herb with lengthy, thin leaves that contains myrcene (an aromatic compound) as well as citral and geraniol (antimicrobial compounds). Therefore, identifying an appropriate drying method for this plant is crucial for maintaining aromatic and antimicrobial compounds and enhancing the shelf life of the product. This investigation seeks to assess the influence of various drying tactics involving hot air at temperatures of 40, 50, and 60 °C, infrared radiation at intensities of 0.5, 0.6, and 0.8 $\mathrm{W}/{\mathrm{cm}}^{2}$, sequential hot-air/infrared, as well as simultaneous hot air-infrared, on the drying mechanism, color, appearance, yield, and essential oil constituents of lemongrass leaves, with the objective of enhancing the marketability of the product. The essential oils of lemongrass were extracted through the process of hydro-distillation, and subsequently, the volatile compounds present were analyzed using Gas Chromatography—Mass Spectrometry (GC–MS). The findings indicated: (a) The most appropriate technique for preserving optimal color quality of lemongrass leaves was through the application of hot air drying solely at a temperature of 60 °C; (b) To optimize the retention and amplification of the essential oil content in lemongrass, our study recommends the employment of a simultaneous hybrid drying technique involving hot air drying at a temperature of 50 °C in conjunction with infrared drying set at a radiation intensity level of 0.6 $\mathrm{W}/{\mathrm{cm}}^{2}$; and (c) The data analysis demonstrated that in order to achieve elevated levels of volatile compounds, specifically neral and geranial, infrared drying with a radiation intensity of 0.6 and 0.8 $\mathrm{W}/{\mathrm{cm}}^{2}$, respectively, was found to be optimal.

## Introduction

Lemongrass, also known as *Cymbopogon citratus*, is a well-known and high-value aromatic herb from the *Poaceae* family that is commonly referred to as lemongrass due to its lemony odor. The lemon-like fragrance exhibited by this herb can be attributed to its elevated quantity of citral^1^. Alternative terminologies for lemongrass include oil grass, silky heads, and citronella grass^2^. Lemongrass, a plant of great importance, boasts a vast variety of around 120 types, predominantly found in warm and temperate regions across the globe. Furthermore, the reason for cultivating this plant on a large scale is due to its multifarious applications in diverse sectors such as pharmaceuticals, cosmetics, food production, flavors, and agriculture^1, 3^. The medicinal applications of lemongrass are diverse, encompassing the management of nervous and gastrointestinal disruptions, while additionally offering therapeutic effects as an antispasmodic, antimicrobial, and anticancer agent^4^. Recent research has revealed that the plant possesses numerous pharmacological advantages, such as analgesic, anti-inflammatory, antipyretic, diuretic, and sedative effects^5^. The supply of fresh plants on a global scale while preserving their quality poses a multifarious challenge. The implementation of drying techniques provides a solution for preserving quality as herbs that are dried exhibit a slower rate of quality degradation compared to fresh herbs. Additionally, dried herbs facilitate storage and transportation, thereby presenting a more controllable alternative^6, 7^. The utilization of drying is an established and universal procedure for safeguarding and upholding the essential bioactive constituents of herbs throughout the postharvest phase^8^. The research conducted on the influence of diverse drying techniques upon medicinal and aromatic plants have indicated a pronounced effect of the drying methods on the yield and the volatile compositions of essential oil of herbs^9–13^. Hot-air drying, also called convection drying, is considered to be an extremely effective method for prolonging the shelf life of various products^2^. Infrared drying presents various notable advantages over hot-air drying. Infrared drying has been shown to enhance the quality of various types of dried agricultural crops, such as fruit, vegetables, and cereals, while also reducing the duration of the drying process^14, 15^. In addition to its superior rates of drying, infrared drying is advantageous due to its energy conservation^16, 17^.

The use of hybrid-drying techniques has resulted in improved quality of herbs that have been dried, most notably, significant advancements in color and aroma^18^. In contemporary times, combining infrared drying with hot air drying method is considered more efficient than using merely radiation or heating techniques, because it produces a synergistic effect. Although there are currently limited reports available on how lemongrass dries, there is no scholarly investigation regarding the influence of infrared drying and also hybrid hot air-infrared drying methods on lemongrass leaves. This research seeks to assess the effectiveness of various drying techniques, namely hot air drying at 40, 50, and 60 °C, infrared drying at 0.5, 0.6, and 0.8 $\mathrm{W}/{\mathrm{cm}}^{2}$ intensities, and a combination of both procedures. The main aim of this study is to evaluate the difference in color between dried and fresh lemongrass leaves on a global scale. Additionally, the study aims to identify the most effective method of drying lemongrass leaves to obtain a greater yield of essential oil and necessary volatile compounds, specifically the isomers of the aldehyde citral (geranial and neral), known as citral a and citral b in geometric terms, respectively.

## Materials and methods

### Plant material

**Figure 1:**
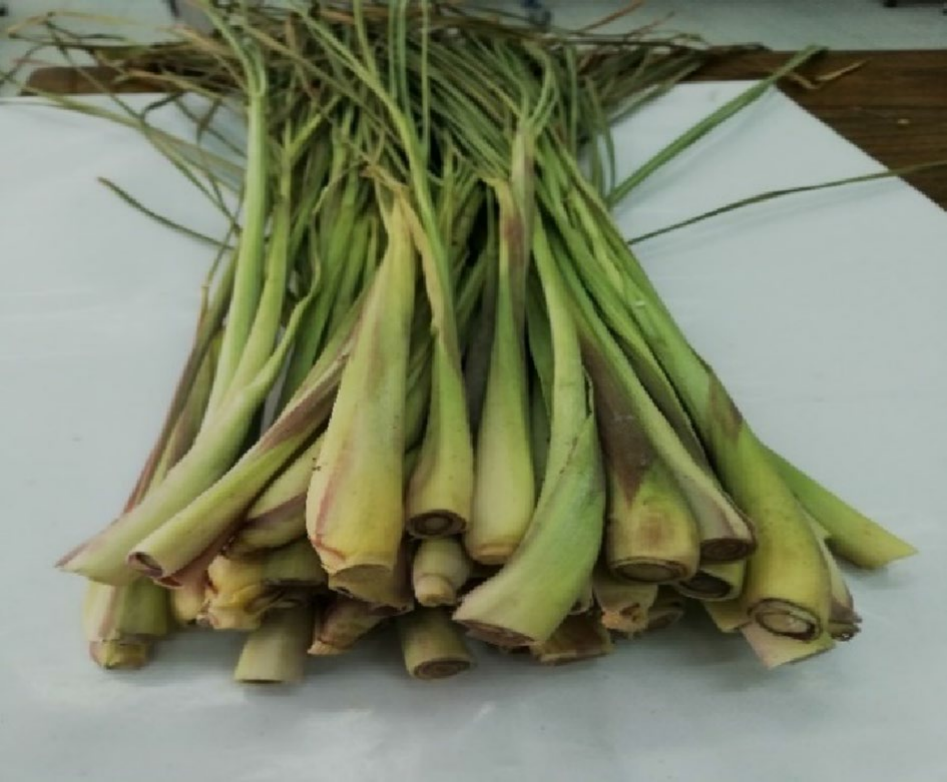
The lemongrass.

**Figure 2:**
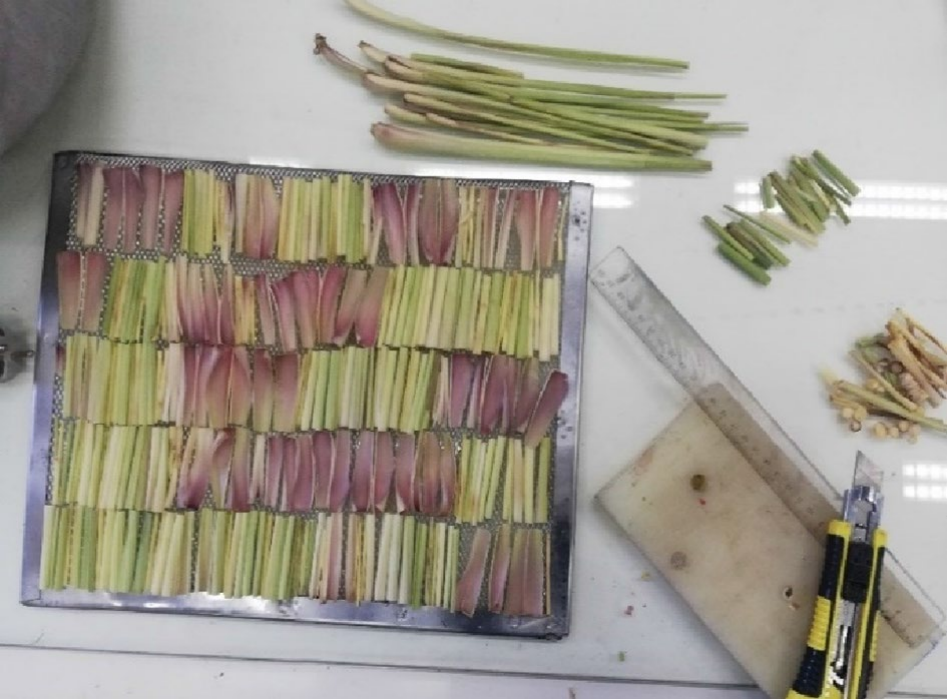
The chopped samples.

Fresh leaves of lemongrass were acquired from a farm located in a village situated in Hormozgan province of Iran (Fig. 1). Initially, fresh leaves with uniform physical characteristics were manually selected and cleaned to become devoid of any dirt and dust, then the leaves were sectioned into 15 cm lengths and subsequently diced into 5 cm pieces, as demonstrated in Fig. 2. This procedure was carried out using a sanitized and sharp carpet cutting tool^19^.

### Methods

#### Moisture content

The moisture content of the samples was determined using the Standard Method of the Association of Official Analytical Chemists (AOAC)^20^. The study involved the preparation of lemongrass samples, wherein approximately 20g of fresh chopped lemongrass prior to drying and about 5 g of dried chopped pieces after the drying process were subjected to an oven dryer, set at a constant temperature of 80.0 ± 2.0 °C, for a duration of 48 h. The samples were placed on an aluminum foil atop the oven dryer tray. Subsequently, the weight of the samples was measured using a digital balance with a minimum count of 0.01 mg, upon their removal from the oven and then the initial and final moisture content of the samples were obtained through the use of Eq. 1 (Eq. 1):

1 $$M_{O} = \frac{{m{}_{1} - m_{2} }}{{m_{1} }}*100$$

In this equation, the parameter denoted as ${M}_{0}$ is representative of the percentage of moisture content of the sample expressed as a percentage (%), whereas ${\mathrm{m}}_{1}$ and ${\mathrm{m}}_{2}$ represent the weight of the sample before and after the drying process, respectively, as measured in grams (g).

#### Drying procedure

**Figure 3:**
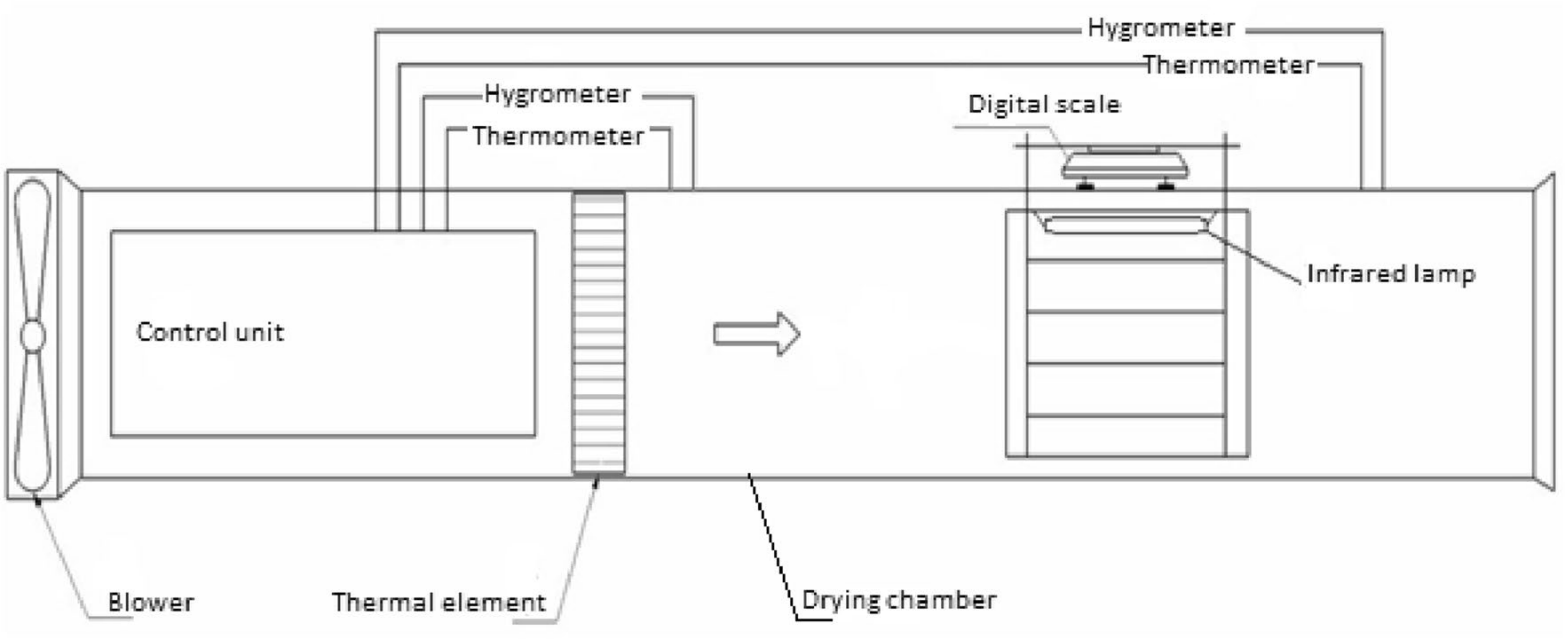
Schematic diagram of hybrid drying system.

The present study employed a laboratory-scale hybrid dryer, combining hot air and infrared technologies, to conduct drying experiments utilizing each technology independently as well as in conjunction with one another (Fig. 3). In all drying procedures, first of all, the dryer was set at Specific intensity of infrared radiation or temperature (see Sections “Hot Air Drying” and “Infrared Drying”). Upon attaining steady-state conditions, a thin layer of the samples consisting of 100 g was subsequently placed onto the tray dryer. The weight of samples was measured via a pre-calibrated digital balance at 3 min intervals throughout the drying process and recorded as entries in an Excel document through an RS232 connection connected to a computer unit. The cessation of each drying experiment was determined upon reaching the point at which the moisture content of the respective samples attained 10–12%.

##### Hot air drying

The study utilized the hot air-drying technique at three discrete temperatures of 40 °C, 50 °C, and 60 °C while maintaining a constant air velocity of 1 ms^−1^. The research was conducted thrice to ensure rigor and validity^2^.

##### Infrared drying

The implementation of the infrared drying process underwent execution across three distinct levels of intensity of infrared radiation. The intensities examined for this study were 0.5, 0.6, and 0.8 $\mathrm{W}/{\mathrm{cm}}^{2}$. Each of these intensities was replicated three times in order to ensure reliable results. The distance between the infrared lamp and the tray was 28 cm, determined from preliminary trials.

##### Hybrid drying

The current study identified the most favorable temperature for hot air drying and the most optimal radiation intensity for infrared drying. These parameters were determined based on their ability to preserve the highest quantity of essential oil and retain the optimal color quality of the dried lemongrass leaves. The selected conditions with these optimal parameters were deemed suitable for use in the hybrid drying method. Subsequently, the combination of hot air drying and infrared drying methods, was carried out in the following manner:

1. Simultaneous drying by hot air (50 °C) and infrared (0.6 $\mathrm{W}/{\mathrm{cm}}^{2}$)
2. Two-stage sequential hot-air (50 °C) and infrared (0.6 $\mathrm{W}/{\mathrm{cm}}^{2}$) drying (firstly, hot air drying until 50% of the moisture content of the product was removed and then continued with infrared until the end of drying).
3. Two-stage sequential infrared (0.6 $\mathrm{W}/{\mathrm{cm}}^{2}$) and hot-air (50 °C) drying (Firstly, infrared drying until 50% of the moisture content of the product was removed and then continued with hot air until the end of drying).

#### Colorimetric parameters of lemongrass leaves

Prior to and following the drying process, five distinct leaves situated at five discrete locations within the tray were chosen at random and subsequently subjected to photographic capture through utilization of the Hunter laboratory chamber (five replications). The purpose of this procedure was to ascertain the Colorimetric parameters (the L*, a*, and b* values) via employment of the Matlab software. Lightness (L*), Redness (a*), and Yellowness (b*) represent the three distinct attributes used to describe color in the CIELAB color space. Subsequent to obtaining the color parameter values for both the fresh and dried samples, the total color difference, commonly referred to as ΔE, was determined utilizing the following equation (Eq. 2):

2 $$\Delta {\text{E}} = \sqrt[2]{{\left( {{\text{l}}^{*}_{0} - {\text{l}}^{*}_{{\text{i}}} } \right)^{2} + \left( {{\text{a}}^{*}_{0} - {\text{a}}^{*}_{{\text{i}}} } \right)^{2} + \left( {{\text{b}}^{*}_{0} - {\text{b}}^{*}_{{\text{i}}} } \right)^{2} }}$$

where the index “_0_” refers to the color of fresh leaves and the index “i” in the equation refers to the color of dried leaves.

#### Extraction of essential oil from lemongrass leaves

After each drying method, a total of 50 g of the lemongrass leaves that had been dried were utilized in an essential oil extraction process. This process was carried out using a hydro-distillation approach employing a Clevenger-type apparatus under a controlled temperature of 60 °C^21^. The duration of this process was between 3 and 4 h (until the essential oil volume remained constant)^22, 23^. The essential oil content (Yield) in the lemongrass was calculated by using Eq. 3^24^:

3 $${\text{Yield}} \left( \% \right) = \frac{{{\text{Essential oil obtained }}\left( {{\text{ml}}} \right)}}{{{\text{Total weight of dried sample}} \left( {\text{g}} \right)}} \times 100$$

#### Gas chromatography-mass spectroscopy analysis of essential oil

The essential oil of lemongrass was subjected to gas chromatography-mass spectroscopy (GC/MS) in order to undertake the process of analysis and identification of the percentages of volatile compounds. The present study utilized an Agilent instrument with model number 19091S-433 to perform GC–MS analyses. The system was equipped with an HP-5MS capillary column measuring 30 m in length and 0.25 mm in diameter. The given column exhibited a film thickness measuring 0.25 µm. The temperature of the oven was varied within the range of 70–240 °C at a constant rate of 7 °C/min, while the transfer line temperature remained at 240 °C. The carrier gas utilized in the experiment was helium with a flow rate of 1 ml/min, and a split ratio of 1:35 was employed. Ionization energy was set at 70 eV, and the mass range analyzed was within 2–800 a.m.u.

#### Statistical analysis

In the present investigation, IBM SPSS Statistics version 26 software was employed for conducting statistical analysis. All comparisons underwent statistical analysis through the application of a one-way analysis of variance (ANOVA). The existence of significant differences between treatment means was subsequently determined by means of the Least Significant Difference (LSD) test, with a significance level set at *p* < 0.05. All graphs were generated through the utilization of the Excel software program.

### Ethical approval

Authors confirm that the required permissions to collect Lemongrass were obtained.

Authors confirm that the use of plants in the present study complies with international, national and/or institutional guidelines.

## Results

### Lemongrass leaf drying

**Figure 4:**
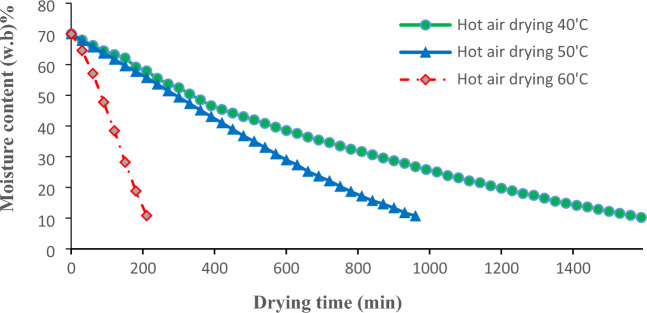
Hot-air drying curves graph of lemongrass leaves at various temperatures.

**Figure 5:**
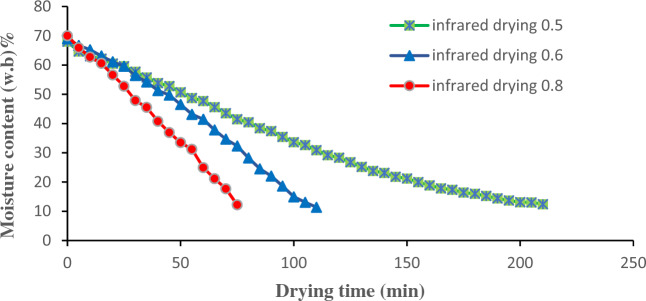
Infrared drying curves graph of lemongrass leaves at various intensities of infrared radiation.

**Figure 6:**
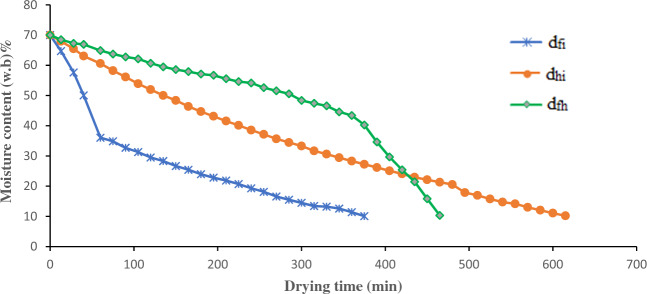
Hybrid drying curves graph of lemongrass leaves. ${\mathrm{d}}_{\mathrm{hi}}:$ Simultaneous drying by hot air and infrared, ${\mathrm{d}}_{\mathrm{fi}}:$ Drying by infrared drying first, until 50% of the moisture content of the product was removed, and then drying by hot air drying, ${\mathrm{d}}_{\mathrm{fh}}:$ Drying by hot air drying first, until 50% of the moisture content of the product was removed, and then drying by infrared drying.

The drying curve graphs of lemongrass leaves were analyzed in this research while utilizing various methods of drying. Based on the drying curves, it was observed that the moisture content decreased from 70.0 ± 3% (wet basis) to a final range of 10–12% (wet basis) for all the methods that were studied. However, it was observed that the drying times varied significantly, with hot air drying taking 210–1590 min, infrared drying taking 75–210 min, and hybrid drying taking 375–615 min (Figs. 4, 5 and 6). The drying process depicted in all curves differs from the standard three-phase drying process (initial, constant-rate, and falling-rate) as it only shows two stages. Based on contemporary research, the phase of constant rate takes place promptly during the drying operations of fruits and vegetables, thus this stage is not present in these processes^2, 25^. The samples of lemongrass were dried from the primary level of moisture content amounting to 70% (the initial phase) until they reached an ultimate level of moisture content of 10–12% during the falling-rate phase. The primary objective of achieving the previously mentioned limit of ultimate moisture content is to effectively retain the crucial constituent of the herb, namely the essential oil. In the event of exceeding these levels of moisture content (10–12%), an excessive drying of the plant ensues, thereby causing a significant depletion of the essential oil content^24, 26, 27^. Moreover, due to these amounts of ultimate moisture content obtained, there is a greater probability for an extended shelf life of the desiccated samples^28^.

It has been observed that when the drying air temperature gets hotter, the surface of plant gets dry faster and the moisture goes from the center to the surface more quickly. When the water evaporates quicker, it also takes less time for samples to dry^2^. Figure 4 shows that when the temperature increased from 40 to 60 °C, the drying time decreased significantly. Such observed phenomenon is caused by an increase in thermal drying force within the sample, which can be linked to the rise in temperature induced by the said drying method. The drying process of lemongrass leaves was carried out at various temperatures, namely 40, 50, and 60 °C, ultimately reaching a moisture content of 10–12%.The duration of the process was recorded at 1590, 960, and 210 min, respectively, for the aforementioned temperatures. Mujaffar and John^29^, two researchers, reported that by raising the temperature to 60 °C from 40 °C, they were able to reduce the drying time of lemongrass leaves. Unfortunately, the utilization of hot air drying did not result in considerable enhancement of the drying process, with the exception of when the temperature was adjusted to 60 °C. Similar conclusions were reached by Kohout et al.^30^ and Xu et al.^31^, in relation to the influence of temperature.

Utilizing infrared drying can significantly enhance the rate at which wet samples dry while also enhancing the overall quality of the end products^31^. The reason for this phenomenon is due to the fact that the infrared dryer has the ability to penetrate wet substances and distribute heat and temperature. Furthermore, it possesses enhanced power density as opposed to the hot air dryer. Alternatively, infrared drying exhibits greater energy efficiency in comparison to hot air drying^18^. Consequently, infrared drying has been shown to be a preferable option for enhancing the efficacy of drying processes^32^. The data presented in Fig. 5 indicate a notable reduction in the drying time as the level of infrared radiation intensity escalates. The research evaluated the drying times of infrared drying at different radiation intensities, namely 0.5, 0.6, and 0.8 $\mathrm{W}/{\mathrm{cm}}^{2}$ which were found to be 210, 110, and 75 min, respectively. These findings are consistent with the analogous observation made by Sharma et al.^33^.

As depicted in Fig. 6, the process of drying resulted in a conventional pattern of diminishing moisture content in the leaves, characterized by an initial swift decline, and subsequent gradual diminishment towards a state of equilibrium. The rate at which samples dries is related to the temperature at which it is being dried. The drying process speeds up as the temperature increases. Alternatively, when the drying temperature goes up, the time needed for drying goes down. Figure 6 illustrates that the plant was dried within 615 min when both infrared and hot air were utilized simultaneously. Meanwhile, it took 375 min to dry using infrared first followed by hot air, and 465 min when hot air was applied first, followed by infrared. These results match the results found by Belghit, et al.^34^. Moreover, Heber^35^and his team carried out research on potatoes and carrots in the same manner. By combining infrared drying and hot air drying, the scientists reported a considerable reduction in the drying time compared to using hot air drying alone^25, 35^. Industrial food drying greatly relies on the significance of time. To minimize it, an alternative approach is to opt for infrared drying rather than other methods such as hot air drying. Among the various methods investigated, it was determined that infrared drying procedures, which have the shortest drying time, exhibit greater efficiency than alternative methods. The observed high efficiency of infrared drying in this context may be attributed to its ability to effectively address challenges related to the thickness problem of lemongrass slices while drying. The leaves heat up quickly through the vibration and friction caused by infrared radiation easily passing through herbs and stimulating water molecules^36^. Consequently, this makes things dry faster and cuts down on how long it takes to dry. The outcomes coincide with the findings of a study carried out by Huang et al.^37^, which focused on the implementation of far-infrared radiation in the drying procedure of *Stevia rebaudiana* leaves.

### Assigning the best conditions for combined drying

The color of food is widely recognized as an important quality parameter that consumers carefully consider prior to purchasing any food product. According to observations, there is a correlation between the appearance quality of a plant and the extent of color difference between fresh and dried herbs. Basically, if the discrepancy in color is minimized, it implies that the plant is of superior quality in terms of appearance and color acceptability. In this study, an investigation was conducted to assess how the utilization of infrared and hot air drying techniques influences the color and yield of essential oil in lemongrass samples using analysis of variance and mean comparisons. The goal of the investigation was to establish the optimal radiation intensity of infrared and temperature of hot air for hybrid drying. The findings of the analysis are presented in Table 1. It can be noted from the findings that there was a clear dissimilarity in the yield of essential oil derived from lemongrass slices when subjected to drying through infrared versus hot air methods. However, the effect of using infrared and hot air for drying on the total color difference was determined to be statistically insignificant (*p* > 0.05). According to Table 1, infrared drying at a radiation intensity of 0.6 $\mathrm{W}/{\mathrm{cm}}^{2}$ yielded the highest yield of essential oil.

**Table 1:** Impact of infrared and hot-air drying procedure on the yield of essential oil and the total color difference of lemongrass herb.

		Essential oil yield	∆E
Infrared drying	0.5 $\mathrm{W}/{\mathrm{cm}}^{2}$	1.47 ± 0.45^b^	6.95 ± 2.49
	0.6 $\mathrm{W}/{\mathrm{cm}}^{2}$	1.85 ± 0.62^a^	5.99 ± 1.97
	0.8 $\mathrm{W}/{\mathrm{cm}}^{2}$	1.35 ± 0.10^b^	7.28 ± 1.30
Hot air drying	40 °C	1.56 ± 0.60^b^	6.61 ± 2.74
	50 °C	2.09 ± 0.25^a^	8.19 ± 1.74
	60 °C	1.38 ± 0.14^b^	4.75 ± 1.24

Means followed by the same letter in each group at the same column did not share significant differences at *p* < 0.05.

Considering hot air drying method, the highest efficiency of essential oil was achieved by utilizing hot air drying at 50 degrees Celsius, as demonstrated in Table 1. The main purpose of this investigation is to ascertain the optimal conditions for preserving essential oil and ensuring lemongrass long-lasting visual attractiveness. The findings imply that when the temperature is 50 °C and the radiation intensity is 0.6 $\mathrm{W}/{\mathrm{cm}}^{2}$, the process of combined hot air-infrared drying is most effective in maintaining a greater efficiency of essential oil. Therefore, these conditions are recommended for the aforementioned purposes.

### Impact of drying tactics on the color of lemongrass herb

Table 2 exhibits the color parameters (L*, a*, b*) and the total color difference (∆E) between newly harvested lemongrass samples and samples that were subjected to drying techniques involving hot air, infrared, and a combined drying. A statistically noticeable dissimilarity (*p* < 0.05) was observed in the color of samples when subjected to various drying techniques. The L*, a*, and b* quantities of dried leaves were observed to range from 51.38 to 58.82, − 3.04 to 3.95, and 27.21 to 31.70, respectively. In overall terms, compared to newly-harvested leaves, the various drying procedures led to a decrease in the lightness and yellowness, while simultaneously elevating the redness. The probable cause for the decrease in L* quantities may be attributed to the generation of dark pigments as a result of the Maillard reaction^38^. The outcomes attained demonstrate that the samples subjected to drying using hot air and combined techniques exhibited comparatively greater L* quantities in contrast to samples dried using infrared drying, respectively, thereby implying that aforementioned techniques possess the potential to yield products with a brighter appearance. The phenomenon of decreased quality or elevated a* quantities in dried samples relative to their newly-harvested counterparts, particularly when exposed to higher temperatures, have been ascribed to the occurrence of Maillard reactions, a reduction in chlorophyll content and essential oils, thereby culminating in the production of a reddish-brown material^29, 39^. Samples subjected to combined and infrared drying procedures exhibited comparatively lower a* quantities in contrast to samples dried via hot air drying, thereby suggesting that aforesaid drying techniques have the potential to yield brighter green products. Furthermore, the variation in b* quantities between dry and newly-harvested leaves indicates a decline in the level of plant yellowness despite temperature's direct influence on the desired factor in all methodologies. Implementing such strategies can make the plant more desirable and accepted in the market. These findings align with the analogous observations made by Xu, et al.^31^.

**Table 2:** Impact of various drying techniques on the colorimetric parameters of lemongrass herb.

		L*	a*	b*	∆E
Fresh leaves	–	58.82 ± 3.09	− 3.04 ± 1.83	31.70 ± 2.36	–
Hot air drying	40 °C	56.47 ± 2.35	− 1.77 ± 2.49	27.21 ± 0.65	6.61 ± 1.09^ab^
	50 °C	56.33 ± 1.62	0.32 ± 2.39	28.23 ± 2.48	8.19 ± 2.04^ab^
	60 °C	55.94 ± 1.44	− 1.82 ± 4.44	30.02 ± 0.83	4.75 ± 2.06^b^
Infrared drying	0.5 $\mathrm{W}/{\mathrm{cm}}^{2}$	53.36 ± 3.41	1.12 ± 1.73	27.82 ± 1.97	6.95 ± 1.32^ab^
	0.6 $\mathrm{W}/{\mathrm{cm}}^{2}$	51.38 ± 5.60	3.95 ± 2.62	28.01 ± 3.88	5.99 ± 2.75^ab^
	0.8 $\mathrm{W}/{\mathrm{cm}}^{2}$	54.30 ± 1.56	2.40 ± 2.34	28.73 ± 1.38	7.28 ± 1.25^ab^
Combined hot air-infrared drying	${\mathrm{d}}_{\mathrm{hi}}$	56.99 ± 3.74	− 0.46 ± 1.96	27.75 ± 2.91	8.51 ± 1.81^a^
	${\mathrm{d}}_{\mathrm{fh}}$	56.13 ± 0.79	− 0.94 ± 1.21	27.41 ± 2.88	7.01 ± 1.99^ab^
	${\mathrm{d}}_{\mathrm{fi}}$	54.27 ± 1.91	− 0.08 ± 0.34	28.96 ± 2.30	6.47 ± 1.58^ab^

L*: Lightness, a*: Redness/greenness, b*:Yellowness/blueness, ∆E: the global color difference between fresh and dried samples, ${\mathrm{d}}_{\mathrm{hi}}:$ Simultaneous drying by hot air and infrared, ${\mathrm{d}}_{\mathrm{fh}}:$ Drying by hot air drying first, until 50% of the moisture content of the product was removed, and then drying by infrared drying, ${\mathrm{d}}_{\mathrm{fi}}:$ Drying by infrared drying first, until 50% of the moisture content of the product was removed, and then drying by hot air drying. Values with the different letters within each column indicate significant differences (*p* < 0.05).

Elevated temperature and prolonged drying have been found to induce degradation of color, as evidenced by the findings of Senadeera et al.^40^. Based on the total color difference (∆E) calculated using newly-harvested leaves and presented in Table 2, the optimal technique for preserving the color of lemongrass leaves entails subjecting them to hot air drying at 60 °C, as evidenced by the lowest amount, which is 4.75. In a research conducted by Xu et al.^31^, an investigation was made on the qualitative alterations of chrysanthemum cake under various drying conditions, namely hot air drying (HAD), combined infrared and hot air drying (IR-HAD), and sequential IR-HAD followed by HAD (IR-HAD + HAD). It was determined that the sequential procedure (IR-HAD + HAD) yielded superior outcomes in terms of preserving the color of the product.

### Impact of drying techniques on the yield of essential oil of lemongrass herb

**Figure 7:**
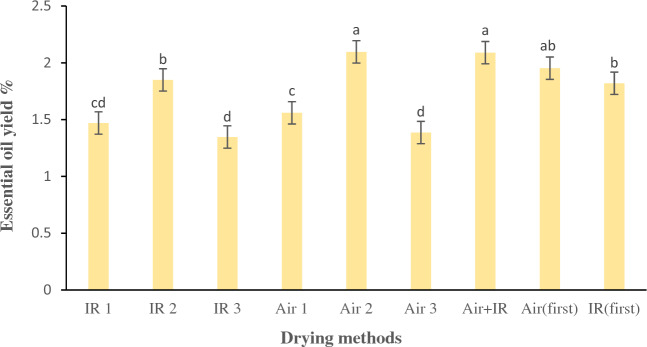
Mean comparisons of the impact of the drying procedures on the yield of essential oil of lemongrass. Bars with the same small letter did not share significant differences at *p* < 0.05. IR 1: infrared drying with radiation intensity of 0.5 W/cm^2^. IR 2: infrared drying with radiation intensity of 0.6 W/cm^2^. IR 3: infrared drying with radiation intensity of 0.8 W/cm^2^. Air 1: hot air drying at the temperature of 40 °C. Air 2: hot air drying at the temperature of 50 °C. Air 3: hot air drying at the temperature of 60 °C. Air + IR: Simultaneous drying by hot air and infrared drying. Air (first): drying by hot air drying first, until 50% of the moisture content of product was removed, and then drying by infrared drying. IR (first): Drying by infrared drying first, until 50% of the moisture content of product was removed, and then drying by hot air drying.

The essential oil of lemongrass encompasses numerous applications, including its employment as a fundamental feedstock in the process of ionone synthesis, which is utilized for the synthesis of some fragrant compositions and vitamin A. Additionally, this oil has been found to serve as a prominent replacement for "cod liver oil" in contemporary usage^24^. Figure 7 presents the data on the efficiency of essential oil extracted from dried leaves. The results indicate that the efficiency of pale-yellow essential oil, acquired through varied drying procedures, ranged from 1.34 to 2.09%. Noticeable dissimilarities (*p* < 0.05) were noticed among the diverse drying techniques utilized in correlation to the yield of essential oil. Caputo, et al.^41^ reported that the drying method has an impact on quantitative and qualitative oil features. Sellami, et al.^42^ found that when sage plants are dried, they produce more essential oils compared to when they are newly harvested. As per the findings illustrated in Fig. 7, both samples underwent hot air drying at 50 °C and those subjected to hybrid drying (Simultaneous drying) achieved the highest yields of essential oil at 2.09%. It is noteworthy to mention that there was no statistically noticeable discrepancy between these two figures of essential oil yield. The greater yield of essential oil observed in these aforesaid procedures is due to the loss of the minimum volatile component of essential oil during the process of drying. What was observed by Kumar et al.^24^ is consistent with the outcomes. Certain plant species possess essential components located on their leaves. The vulnerability of these plants to temperature variations can result in substantial depletion of their essential oil content. The reason for this occurrence is that the oil-producing glands rupture, causing the oil to rapidly evaporate^43^. The protracted drying process engenders heightened levels of evaporation, leading to a diminution in the amount of essential oil. Additionally, this process results in squandered energy and time. Consequently, it is notable that the utilization of the hybrid drying technique, which involves the simultaneous application of hot air at 50 °C and infrared radiation at a intensity of 0.6 $\mathrm{W}/{\mathrm{cm}}^{2}$, results in a shorter drying time (around ten and half hours) relative to the drying process characterized by exclusive hot air drying at 50 °C (16 h). Hence, it can be inferred that the implementation of the simultaneous hybrid drying approach for lemongrass leaves is more appropriate and advisable in terms of saving time and acquiring a superior yield of essential oil. The aforementioned findings demonstrate conformity with the outcomes acquired by Hanaa et al.^1^.

### Investigation of essential oil compounds

The analysis of essential oils extracted from dried leaves of lemongrass, employing various drying techniques, was conducted via the utilization of gas chromatography/mass spectrometry. By analyzing the data, the constituents of the essential oil found in each treatment were recognized and the efficiencies associated with them were computed. The relative percentage of important volatile components discovered in lemongrass essential oils is exhibited in Table 3. These compounds are listed in order based on their retention indices (RI) on the HP-5MS column. The desiccation technique yielded a noteworthy impact on the proportion of disparate constituents. The present research revealed that the essential oil of dried plant slices, obtained through various drying techniques, comprise a total of seventeen major constituents which account for 94.75–58.80% of the oil's total composition. The principal and most important components of the essential oils derived from dried lemongrass samples through the process of hot air, infrared, and hybrid drying, were identified as geranial (50.43%, 58.18%, and 33.94% respectively) and neral (39.22%, 30.30%, and 30.30% respectively) as presented in Table 3; The evaluation of lemongrass quality is commonly based on the geranial and neral quantities^1^. These results match with the discoveries made by Hanaa et al.^1^; Morsy^10^; Omidbaigi et al.^13^; Sefidkon et al.^44^. The outcomes reveal that infrared drying of lemongrass herb leads to notably elevated levels of geranial and neral in the resulting oil, surpassing the levels achieved through other drying techniques.

**Table 3:** Essential oil components yield of lemongrass samples as affected by various drying procedures.

No	Compound	RI	${\mathrm{IR}}_{0.5\,\mathrm{ W}/{\mathrm{cm}}^{2}}$	${\mathrm{IR}}_{0.6\,\mathrm{ W}/{\mathrm{cm}}^{2}}$	${\mathrm{IR}}_{0.8\,\mathrm{ W}/{\mathrm{cm}}^{2}}$	${\mathrm{air}}_{40\,^\circ \mathrm{C}}$	${\mathrm{air}}_{50\,^\circ \mathrm{C}}$	${\mathrm{air}}_{60\,^\circ \mathrm{C}}$	${\mathrm{d}}_{\mathrm{hi}}$	${\mathrm{d}}_{\mathrm{fh}}$	${\mathrm{d}}_{\mathrm{fi}}$
1	Beta-myrcene	991	1.77 ± 0.77	1.33 ± 0.28	1.13 ± 0.99	1.71 ± 0.26	1.37 ± 0.78	2.86 ± 1.07	–	–	–
2	Beta-ocimene	1044	–	–	–	–	0.12 ± 0.07	0.72 ± 0.53	–	–	–
3	Alpha-ocimene	1050	0.20 ± 0.13	0.11 ± 0.01	0.61 ± 0.41	0.45 ± 0.49	0.15 ± 0.14	1.07 ± 1.04	0.70 ± 0.79	–	–
4	Linalool	1102	0.64 ± 0.43	1.87 ± 1.09	0.43 ± 0.70	0.48 ± 0.64	1.07 ± 0.72	1.46 ± 0.35	1.09 ± 0.57	1.09 ± 0.99	0.40 ± 0.20
5	Citronella	1154	–	–	–	0.37 ± 0.54	–	–	–	–	–
6	Beta-citronellol	1227	0.12 ± 0.08	0.52 ± 0.26	1.16 ± 0.89	0.90 ± 0.79	1.05 ± 1.27	0.45 ± 0.43	0.82 ± 0.71	1.11 ± 0.87	–
7	Nerol	1230	–	0.15 ± 0.13	–	–	–	–	–	–	0.27 ± 0.13
8	Neral	1244	39.22 ± 0.99	45.25 ± 0.94	38.55 ± 0.97	39.50 ± 1.53	39.84 ± 0.89	44.56 ± 0.99	34.39 ± 1.05	30.30 ± 0.96	33.87 ± 1.03
9	Geraniol	1256	–	0.95 ± 0.15	–	2.22 ± 1.16	–	–	3.29 ± 0.81	6.11 ± 0.94	-
10	Gernial	1273	50.43 ± 1.31	42.24 ± 1.05	51.65 ± 0.82	43.59 ± 0.99	41.52 ± 1.16	40.61 ± 0.75	42.27 ± 1.15	33.94 ± 1.00	37.60 ± 0.80
11	Geranyl acetate	1378	–	–	–	1.04 ± 0.82	–	2.04 ± 0.92	–	–	–
12	Beta-farnesene	1442	–	–	–	0.83 ± 0.43	–	–	–	–	–
13	Beta-bisabolene	1514	–	–	–	0.51 ± 0.44	–	–	0.48 ± 0.59	–	–
14	Caryophyllene oxide	1592	–	0.36 ± 0.12	–	–	0.62 ± 0.55	–	–	–	–
15	Viridiflorol	1601	0.35 ± 0.12	0.50 ± 0.43	0.55 ± 0.31	–	0.46 ± 0.40	0.38 ± 0.16	–	0.77 ± 0.42	–
16	Farnesol	1726	–	–	–	2.47 ± 1.18	0.75 ± 0.73	0.60 ± 0.98	4.03 ± 0.97	1.69 ± 0.73	5.01 ± 0.99
17	Trans farnesol	1726	–	–	–	–	–	–	–	0.48 ± 0.68	–
	Sum of compounds		92.73	93.28	94.08	94.07	86.95	94.75	87.07	75.49	77.15
	Sum of monoterpenes		52.4	43.68	53.39	47.16	43.16	47.3	42.97	33.94	37.60
	Sum of monoterpenoids		39.98	48.74	40.14	43.1	41.96	46.47	39.59	38.61	34.45
	Sum of sesquiterpenes		–	–	–	1.34	–	–	0.48	–	–
	Sum of sesquiterpenoids		0.35	0.86	0.55	2.47	1.21	0.98	4.03	2.94	5.01

IR and air stand for infrared drying and hot air drying, respectively. ${\mathrm{d}}_{\mathrm{hi}}:$ Simultaneous drying by hot air and infrared drying, ${\mathrm{d}}_{\mathrm{fh}}:$ drying by hot air drying first, until 50% of the moisture content of the product was removed, and then drying by infrared drying, ${\mathrm{d}}_{\mathrm{fi}}:$ Drying by infrared drying first, until 50% of the moisture content of the product was removed, and then drying by hot air drying.

**Figure 8:**
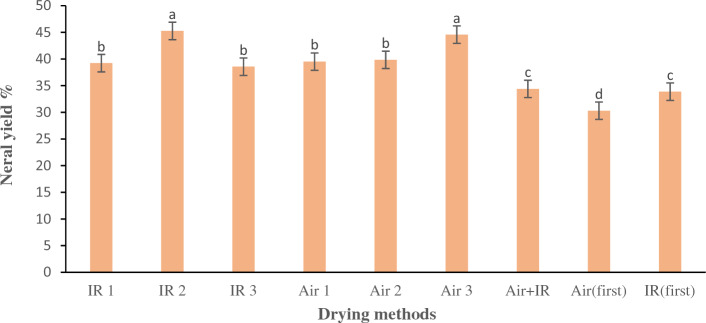
Mean comparisons of the effect of the drying method on the yield of neral obtained from the essential oil of lemongrass. Bars with the same small letter did not share significant differences at *p* < 0.05. IR 1: infrared drying with radiation intensity of 0.5 W/cm^2^. IR 2: infrared drying with radiation intensity of 0.6 W/cm^2^. IR 3: infrared drying with radiation intensity of 0.8 W/cm^2^. Air 1: hot air drying at the temperature of 40 °C. Air 2: hot air drying at the temperature of 50 °C. Air 3: hot air drying at the temperature of 60 °C. Air + IR: Simultaneous drying by hot air and infrared drying. Air (first): drying by hot air drying first, until 50% of the moisture content of product was removed, and then drying by infrared drying. IR (first): Drying by infrared drying first, until 50% of the moisture content of product was removed, and then drying by hot air drying.

**Figure 9:**
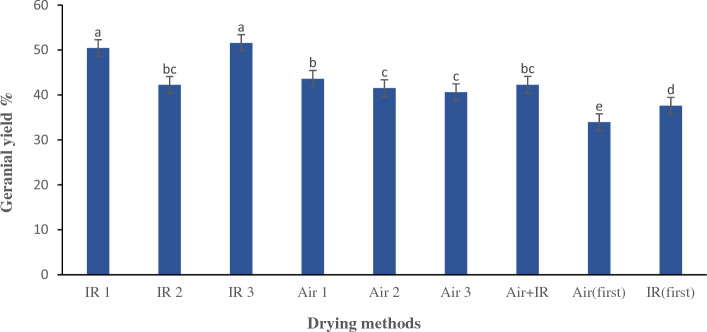
Mean comparisons of the effect of the drying method on the yield of Geranial obtained from the essential oil of lemongrass. Bars with the same small letter did not share significant differences at *p* < 0.05. IR 1: infrared drying with radiation intensity of 0.5 W/cm^2^. IR 2: infrared drying with radiation intensity of 0.6 W/cm^2^. IR 3: infrared drying with radiation intensity of 0.8 W/cm^2^. Air 1: hot air drying at the temperature of 40 °C. Air 2: hot air drying at the temperature of 50 °C. Air 3: hot air drying at the temperature of 60 °C. Air + IR: Simultaneous drying by hot air and infrared drying. Air (first): drying by hot air drying first, until 50% of the moisture content of product was removed, and then drying by infrared drying. IR (first): Drying by infrared drying first, until 50% of the moisture content of product was removed, and then drying by hot air drying.

Certain components were exclusively present in a particular treatment. Trans farnesol (0.48%) was exclusively detected in the ninth treatment (${\mathrm{d}}_{\mathrm{fi}}$). Beta-farnesene and Citronella were detected exclusively in the fourth treatment, which involved the process of hot air drying at a temperature of 40 °C, at percentages of 0.83% and 0.37% respectively. Beta-farnesene is a compound with diverse potential applications, including inhibition of DPPH free radicals, anti-cancer, anti-bacterial, and anti-fungal activity. Further, beta-farnesene has been found to exhibit neuro-protective impacts in rat cortical prime neurons in a dose-dependent manner. Specifically, it inhibits the diffusion of intracellular lactate dehydrogenase (LDH) caused by hydrogen peroxide (${\mathrm{H}}_{2}{\mathrm{O}}_{2}$) and reduces DNA detriment by 47.8%. These findings highlight the promising therapeutic potential of beta-farnesene in the treatment of neurodegenerative diseases^22^. The sum of constituents, monoterpenes and monoterpenoids, present in lemongrass essential oil manifested comparatively greater values in the initial six treatments, as opposed to the remaining treatments. Oils acquire their unique fragrance due to the presence of certain monoterpenes in it. The lemony scent of lemon oil is attributed to a chemical compound known as geranial^45^. Lemongrass contains citral, which is classified as a monoterpene aldehyde. The predominant reason for the intense aroma of lemongrass is mainly because it contains a large amount of citral aldehyde^1^. Neral and geranial are known as isomers of the citral compound, which are regarded paramount constituents of the essential oil of Lemongrass. Citral has various therapeutic uses, including muscle spasm reduction, microbe-preventing capabilities, inflammation reduction, pain relief, and aiding chemotherapy treatments^46–48^. Porto et al. (2014) reported on the potential of citral to mitigate genotoxicity induced by DXR in mouse peritoneal macrophages via its anti-mutagenic activity. Table 3 delineates that the elevated quantity of essential oil extracted from the lemongrass plant is predominantly composed of neral and geranial, wherein their concentrations exhibit variability contingent upon the specific method of drying employed. The significant concentration of aforementioned constituents can be attributed to their elevated vaporization temperature, which this characteristic limits their rate of evaporation considerably throughout the course of the drying proceeding^24^. It can be inferred that the employment of infrared and hot-air drying techniques for lemongrass leaves is more suitable and suggested for the achievement of elevated yields of the principal constituents of essential oil. Notwithstanding numerous other benefits and diverse applications of citral, the elevated quantity of citral yields a concentrated aroma and flavor that is valuable in the production of fragrances for the perfumery industry, as well as soaps, cosmetics, and flavorings for carbonated beverages^24^. To ascertain the optimal technique for procuring a greater quantity of neral and geranial, an analysis of variance and mean comparisons were executed through implementation of the LSD test. Figures 8 and 9 present the mean comparisons pertaining to the impact of the drying technique on the efficiencies of neral and Geranial in the essential oil extracted from lemongrass, respectively.

A statistically significant dissimilarity (*p* < 0.05) was evident between the yield of neral extracted from the essential oil of lemongrass herb that underwent diverse drying techniques. Based on the findings of the mean comparisons (as illustrated in Fig. 8), the high yield of neral was found to be achieved through infrared drying with a radiation intensity of 0.6 $\mathrm{W}/{\mathrm{cm}}^{2}$, which resulted in a yield of 45.25%. In addition, hot air drying at a temperature of 60°C also produced a high yield of neral, with a yield of 44.56%. As formerly alluded to, the protracted drying proceeding engenders increased vaporization, consequentially diminishing the quantity of essential oil, whilst also resulting in a squandering of valuable resources, both temporally and energetically Thus, taking into account the prolonged drying time of 2 and a half hours associated with the hot air drying method at 60 °C in contrast to the comparatively lesser drying time of an hour and a half achieved by the infrared drying technique with a radiation intensity of 0.6 $\mathrm{W}/{\mathrm{cm}}^{2}$, It can be inferred that the latter approach of drying lemongrass leaves is more advisable and optimal in terms of saving time and gaining an elevated yield of neral, a volatile compound of the essential oil. The present findings are found to be consistent with the outcomes derived by Hanaa et al.^1^.

A notable dissimilarity (*p* < 0.05) was observed between the yield of geranial extracted from the essential oil of lemongrass herb subjected to diverse drying procedures. Based on the outcomes of the mean comparisons presented in Fig. 9, it can be inferred that the yield of geranial was found to be highest under the conditions of infrared drying with a radiation intensity of 0.8 $\mathrm{W}/{\mathrm{cm}}^{2}$ (51.56%), followed closely by infrared drying with a radiation intensity of 0.5 $\mathrm{W}/{\mathrm{cm}}^{2}$ (50.43%). Statistical analysis suggests that there is no statistically notable discrepancy between these two conditions. The utilization of the infrared drying technique with a radiation intensity of 0.8 $\mathrm{W}/{\mathrm{cm}}^{2}$, is deemed a preferable and recommended approach for saving time and achieving a high yield of geranial extracted from the essential oil. This deduction arises due to the fact that the drying time was significantly shorter, taking just one hour as opposed to almost three hours in the case of the infrared drying method with a radiation intensity of 0.5 $\mathrm{W}/{\mathrm{cm}}^{2}$. Hanaa et al.^1^ announced that the yield of geranial during the drying process of lemongrass in an oven set at 45°C for a period of 7 h was determined to be 24.37, which is concordant with the amounts gained in this research.

## Conclusion

The study investigated the influence of drying on the drying mechanism, color, yield, and essential oil constituents of lemongrass leaves. Results indicated that there was a significant difference between treatments of each parameter with respect to the drying methods used. It was found that using infrared drying with a radiation intensity of 0.8 $\mathrm{W}/{\mathrm{cm}}^{2}$, hot air at 60 °C and infrared first followed by hot air, led to shorter drying times compared to other methods, taking 55, 150, and 165 min, respectively. Lemongrass samples dried by hot air drying solely at a temperature of 60 °C was the most preferred in color quality. simultaneous hybrid drying technique involving hot air drying at a temperature of 50 °C in conjunction with infrared drying set at a radiation intensity level of 0.6 $\mathrm{W}/{\mathrm{cm}}^{2}$ was found to be most suitable for drying of lemongrass leaves in order to maintain the higher yield of essential oil content. Infrared drying with a radiation intensity of 0.6 $\mathrm{W}/{\mathrm{cm}}^{2}$, and 0.8 $\mathrm{W}/{\mathrm{cm}}^{2}$, was found to be optimal to attain elevated levels of volatile compounds, especially neral and geranial, respectively.

## Data Availability

The datasets used and/or analyzed during the current study available from the corresponding author on reasonable request.
